# The association between lactate, mean arterial pressure, central venous oxygen saturation and peripheral temperature and mortality in severe sepsis: a retrospective cohort analysis

**DOI:** 10.1186/s13054-016-1243-3

**Published:** 2016-03-12

**Authors:** Aletta P. I. Houwink, Saskia Rijkenberg, Rob J. Bosman, Peter H. J. van der Voort

**Affiliations:** Department of Intensive Care, OLVG, Amsterdam, The Netherlands; Department of Intensive Care, AvL hospital, Amsterdam, The Netherlands; TIAS school for Business and Society, Tilburg University, Tilburg, The Netherlands

**Keywords:** Sepsis, Mean arterial pressure, Lactate, Central venous oxygen saturation, Peripheral temperature, Outcome, ICU, Resuscitation

## Abstract

**Background:**

During resuscitation in severe sepsis and septic shock, several goals are set. However, usually not all goals are equally met. The aim of this study is to determine the relative importance of the different goals, such as mean arterial pressure (MAP), lactate, central venous oxygen saturation (ScvO_2_) and central to forefoot temperature (delta-T), and how they relate to intensive care unit (ICU) and hospital mortality.

**Methods:**

In a retrospective cohort study in a 20-bed mixed medical and surgical ICU of a teaching hospital we studied **c**onsecutive critically ill patients who were admitted for confirmed infection and severe sepsis or septic shock between 2008 and 2014. All validated MAP, lactate levels, ScvO_2_ and delta-T for the first 24 hours of ICU treatment were extracted from a clinical database. Logistic regression analyses were performed on validated measurements in the first hour after admission and on mean values over 24 hours. Patients were categorized by MAP (24-hour mean below or above 65 mmHg) and lactate (24-hour mean below or above 2 mmol/l) for Cox regression analysis.

**Results:**

From 837 patients, 821 were eligible for analysis. All had MAP and lactate measurements. The delta-T was available in 812 (99 %) and ScvO_2_ was available for 193 out of these patients (23.5 %). Admission lactate (*p* < 0.001) and admission MAP (*p* < 0.001) were independent predictors of ICU and hospital mortality. The 24-hour mean values for lactate, MAP and delta-T were all independent predictors of ICU mortality. Hospital mortality was independently predicted by the 24-hour mean lactate (odds ratio (OR) 1.34, 95 % confidence interval (CI) 1.30–1.40, *p* = 0.001) mean MAP (OR 0.96, 95 % CI 0.95–0.97, *p* = 0.001) and mean delta-T (OR 1.09, 95 % CI 1.06–1.12, *p* = 0.001). Patients with a 24-hour mean lactate below 2 mmol/l and a 24-hour mean MAP above 65 mmHg had the best survival, followed by patients with a low lactate and a low MAP.

**Conclusions:**

Admission MAP and lactate independently predicted ICU and hospital mortality. The 24-hour mean lactate, mean MAP and mean delta-T independently predicted hospital mortality. A Cox regression analysis showed that 24-hour mean lactate above 2 mmol/l is the strongest predictor for ICU mortality.

## Background

Intensive care clinicians use several concomitant resuscitation goals to guide their treatment, such as arterial and venous pressure, cardiac output, central venous oxygen saturation (ScvO_2_) and serum lactate levels. The Surviving Sepsis Campaign (SSC) guideline is in use worldwide and provides clinicians with clear recommendations on mean arterial pressure (MAP), ScvO_2_ and lactate levels [1]. The targeted MAP of at least 65 mmHg in the SSC guideline has been recently challenged by several studies [2–7]. Some studies do not show improved outcome when a higher blood pressure is achieved [2], but other studies show that a lower mean blood pressure of 50–60 mmHg is associated with a higher incidence of renal failure [3]. For the serum lactate level it is recommended to target normal levels [1]. A lactate level below 4 mmol/l is usually considered acceptable. However, levels in the normal range (below 2 mmol/l) may be associated with better outcomes [8–10]. In addition, it is unclear whether targeting the microcirculation should be a resuscitation goal as well [11, 12]. As a proxy for the microcirculation, the central to toe temperature difference is sometimes used [13, 14].

In the clinical setting a discrepancy between different resuscitation goals sometimes arises where, for instance, either lactate or MAP is abnormal. It is unclear whether all resuscitation goals are equally related to mortality or whether one of them may need more attention than the other. The aim of this study is to gain insight into the association of several resuscitation targets with mortality. We performed a retrospective analysis of several resuscitation goals over the first 24 hours in critically ill patients admitted for severe sepsis or septic shock, and related them to outcome.

## Methods

### Patients

Consecutive critically ill patients were included in a retrospective cohort study over the period January 2008 till January 2014. Patients were included when they were aged 18 years or older, and when they had a confirmed infection on admission and one or more organ failures. To prevent the inclusion of elective surgery patients, we excluded patients with planned admissions. Patients were also excluded when a recent myocardial infarction, chronic renal insufficiency or liver cirrhosis was present. In addition, pregnant patients and patients after cardiac surgery were excluded. Patients were excluded for analysis when no lactate measurements were available at all.

### Setting and therapeutic management

The study was performed at the intensive care unit of Onze Lieve Vrouwe Gasthuis (OLVG), a 20-bed mixed intensive care unit (ICU) of a teaching hospital in Amsterdam, The Netherlands.

Baseline treatment was unchanged during the study period and consisted of broad-spectrum antibiotics aiming at the potential source of infection after blood cultures. In addition, surgical source control was achieved when needed. Fluid resuscitation with crystalloids and norepinephrine and/or dopamine was given where appropriate to achieve MAP to at least 60 mmHg or to a level that was deemed appropriate by the attending physician. Mechanical ventilation was indicated for patients who were in respiratory distress, at the discretion of the attending physician. All septic shock patients received corticosteroids until shock was reversed, usually a 5-day tapering dose of prednisone from 40 to 0 mg per day. Feeding was started on admission and glucose regulation was performed with a nurse-driven protocol to maintain glucose between 6–9 mmol/l. A hemoglobin target of 7–9 g/dl was held according to Dutch guidelines.

### Data collection

Baseline variables such as gender, age, severity of disease (Acute Physiology and Chronic Health Evaluation (APACHE) IV and Sequential Organ Failure Assessment (SOFA)) and treatment data such as mechanical ventilation, renal replacement therapy, vasoactive drugs and fluids were all extracted over the first 24 hours after admission. The data were stored in the Patient Data Management System (PDMS; Metavision, iMDSoft, Tel Aviv, Israel). MAP was measured continuously, but the nurse validated the data at least once per hour at the bedside. All validated MAPs were extracted from the first 24 hours. When invasive arterial pressure measurement was not available, non-invasive measurement was extracted instead. Patients were categorized according to MAP below or above 65 mmHg (admission or arithmetic mean of 24 hours) and shown as MAP(adm) or MAP(24 h) based on the current guideline [1].

Lactate was routinely measured on admission of all patients and at every arterial blood gas analysis (Radiometer, Copenhagen, Denmark). All lactate measurements for the first 24 hours after admission were extracted from the PDMS. Lactate values are shown as admission lactate (lactate(adm)) and as the arithmetic mean of all available measurements over the first 24 hours of ICU stay (lactate(24 h)). Patients were categorized according to lactate(adm) or lactate(24 h) below or above 2 mmol/l. Temperature was routinely measured centrally and on the forefoot to obtain a change in temperature (delta-T) between the central and peripheral location. All available delta-T for the first 24 hours after admission were extracted from the PDMS and shown as admission measurement (delta-T(adm)) and as arithmetic mean of all validated measurements during the first 24 hours after admission (delta-T(24 h)). ScvO_2_ was not routinely measured on admission; data are shown as arithmetic mean of the 24-hour data (ScvO_2_(24 h)). The time between measurements was not taken into account when calculating the arithmetic mean for MAP, lactate, ScvO_2_ and delta-T.

### Ethical statement

The local Medical Ethical Committee of the Onze Lieve Vrouwe Gasthuis approved the study. The need for informed consent was waived because of the retrospective and observational design of the study according to Dutch and European legislation.

### Statistical analysis

Three analyses were made according to a predefined analysis plan: 1) using the admission data within the first hour in the ICU, marked as (adm); 2) calculated mean values over the first 24-hour period in the ICU, marked as (24 h); and 3) according to four groups with either MAP(24 h) below or above 65 mmHg and lactate(24 h) above or below 2 mmol/l.

One database contained the original data and additional databases contained the results of multiple imputations for missing values. The Markov Chain Monte Carlo method (MCMC) for multiple imputation technique was used to obtain these missing data [15]. Multiple imputation was performed for MAP(adm), which was missing in 4.9 % of patients, delta-T(adm), which was missing in 34.6 % of patients, and lactate(adm) which was missing in 16 % of the patients. Baseline variables are described as mean with standard deviation (SD) for variables with a normal distribution. All other variables are shown as median with interquartile range (IQR). Normality was checked by Q-Q plot analyses and skewness and kurtosis tests for normality. Groups were compared with parametric or non-parametric tests where applicable. Nominal variables were tested with Fisher exact test, and continuous variables with the non-parametric Kruskal-Wallis test. All analyses were tested two-sided with a significance level of 0.05. Univariate analyses were made for all variables but multivariate regression analyses were performed with MAP, lactate, and delta-T only because we did not aim to develop an overall prediction model. However, additional backward conditional regression analyses were performed on the model to test the effect of APACHE IV predicted mortality as a measurement of severity of disease as well as with noradrenaline and dopamine dose and fluid balance. ScvO_2_ was not included in the logistic analysis because of the large number of missing data. Multivariate logistic regression analyses with the imputed variables were used to test MAP, lactate and delta-T as independent variables and both hospital and ICU mortality, respectively, as dependent variables. The independent variables were all entered in block 1. These regression analyses were performed with data (adm) and (24 h). Because of the large numbers of missing ScvO_2_ data we performed the analyses without ScvO_2_. To study the effect of time, the consecutive ICU numbers of the patients were entered as well. Multicollinearity of the variables was excluded by correlation check (all correlation coefficients appeared below 0.4). Effect modification was excluded by analysis of interaction terms. Hosmer-Lemeshow goodness of fit and receiver operator characteristic curve (ROC) analyses were made for each regression analysis.

A Cox regression analysis was performed with the four groups based on lactate(24 h) (above or below 2 mmol/l) and MAP(24 h) (above or below 65 mmHg) and length of ICU stay as the dependent value. The lactate threshold of 2 mmol/l was chosen because of the current literature on the relation between lactate and mortality [8–10]. The threshold for MAP was chosen because of current guidelines [1]. All statistical analyses were made using Statistical Package for Social Science (SPSS 21.0 Chicago, IL, USA).

## Results

A total of 837 consecutive patients were included. Lactate measurements were absent in 16 patients, and thus 821 patients were eligible for analysis. The total number of lactate measurements was 3538 in 638 patients (mean 5.45 measurements per patient) for patients with a MAP above 65 mmHg vs. 1096 measurements in 183 patients (mean 5.98 per patient) with blood pressure below 65 mmHg. In these patients the delta-T was available in 812 (99 %) and ScvO_2_ was available for 193 out of the 821 patients (23.5 %).

Table 1 shows the baseline characteristics of all included patients. In addition, the characteristics of the four distinct categories based on MAP(24 h) and lactate(24 h) are shown. The hospital mortality was highest (53.9 %) when lactate(24 h) was above 2 mmol/l and MAP(24 h) below 65 mmHg and mortality was lowest when lactate(24 h) was low and MAP(24 h) was high (15.8 %) (Table 1).

**Table 1:** Baseline characteristics of included patients

	All *N* = 821	Lactate(24 h) <2 mmol/l	Lactate(24 h) <2 mmol/l	Lactate(24 h) ≥2 mmol/l	Lactate(24 h) ≥2 mmol/l	*p* value*
		MAP(24 h) <65 mmHg	MAP(24 h) ≥65 mmHg	MAP(24 h) <65 mmHg	MAP(24 h) ≥65 mmHg	
		*N* = 68	*N* = 406	*N* = 115	*N* = 232	
Male (%)	532/821 (64.8)	45/68 (66.2)	274/406 (67.5)	77/115 (67.0)	136/232 (58.6)	0.15
Age (years)	64.0 (14.4)	68.7 (12.1)	62.8 (14.4)	68.2 (11.3)	62.5 (15.8)	0.001
MAP(adm)	76.9 (21)	64.8 (14)	83.2 (21)	62.6 (14)	76.5 (22)	0.001
MAP(24 h)	72.7 (10)	61.6 (2.9)	77.3 (9.4)	60.2 (4.4)	73.9 (8.1)	0.001
Lactate(adm)	1.9 (1.2–3.5)	1.3 (1.1–1.9)	1.3 (1.0–1.7)	4.2 (2.7–6.6)	3.5 (2.6–5.2)	0.001
Lactate(24 h)	1.8 (1.2–2.8)	1.3 (1.1–1.8)	1.3 (1.1–1.6)	3.9 (2.7–6.2)	2.9 (2.3–4.1)	0.001
ScvO_2_(adm)	66.4 (11)	76.4 (3.3)	68.3 (9.0)	63.9 (11)	64.7 (13)	0.50
ScvO_2_(24 h)	69.3 (9.4)	71.1 (11)	71.3 (8.6)	68.4 (7.6)	67.3 (9.9)	0.063
Delta-T(adm)	6.6 (4.2–9.3)	7.2 (3.2–10)	5.9 (3.9–8.8)	7.4 (5.1–9.7)	7.4 (4.8–9.9)	0.007
Delta-T(24 h)	3.7 (2.8–5.1)	3.4 (2.7–4.9)	3.3 (2.5–4.5)	4.5 (3.3–6.1)	4.0 (3.1–5.8)	0.001
24-h fluid balance (ml)	3366 (1314–5568)	4321 (2160–5939)	2238 (825–3973)	7080 (3671–9795)	3932 1722–6277)	0.001
Mechanical ventilation (%) at any time during ICU stay	627/821 (76)	52/68 (76.5)	304/406 (74.9)	95/115 (82.6)	176/232 (75.9)	0.39
P/F ratio (lowest during ICU stay)	156 (105–226)	169 (117–232)	160 (107–220)	140 (89–202)	166 (105–257)	0.04
Noradrenalin dose^a^ (mg)	0.0 (0–4.2)	0.57 (0–4.8)	0.0 (0–0.85)	7.4 (0.98-19.2)	0.72 (0–6.9)	0.001
Dopamine dose^a^ (mg)	215 (59–469)	402 (230–727)	172 (0–320)	515 (231–912)	219 (62–473)	0.001
History of arterial hypertension	237/821 (28.9)	18/68 (26.5)	131/406 (32.3)	34/115 (29.6)	54/232 (23.3)	0.11
CRRT^b^ (%)	121/821 (14.7)	12/68 (17.6)	22/406 (5.4)	49/115 (42.6)	38/232 (16.4)	0.001
SOFA admission	7.8 (3.7)	8.1 (3.2)	6.5 (2.7)	10.9 (4.5)	8.7 (3.8)	0.001
APACHE IV predicted mortality	0.34 (0.16–0.60)	0.37 (0.19–0.57)	0.25 (0.13–0.45)	0.63 (0.37–0.84)	0.37 (0.18–0.67)	0.001
Length of ICU stay in hours	86.0 (40.0–209)	96.5 (47.8–254)	72 (39.0–189)	120 (64.0–283)	80.5 (36.0–199)	0.013
ICU mortality (%)	146/821 (17.8)	13/68 (19.1)	37/406 (9.1)	51/115 (44.3)	45/232 (19.4)	0.001
Hospital mortality (%)	215/821 (26.2)	18/68 (26.5)	64/406 (15.8)	62/115 (53.9)	71/232 (30.6)	0.001

Values for MAP are given in mmHg, values for lactate in mmol/l, values delta-T in °C and values for ScvO_2_ in %

Groups represent mean lactate and mean MAP over the first 24 hours of ICU admission. Data are given as mean (standard deviation) or median (interquartile range)

*p-value shows differences between all groups

^a^ Cumulative dose over first 24 hours of ICU admission

^b^ At any time during ICU admission, available for 193 patients

*(24 h)* arithmetic mean of all validated measurements during the first 24 hours after admission, *(adm)* at admission, *APACHE* Acute Physiology and Chronic Health Evaluation, *CRRT* continuous renal replacement therapy, *delta-T* difference between central and forefoot temperature, *ICU* intensive care unit, *MAP* mean arterial pressure, *P/F* PaO2 / FiO2 ratio, *ScvO*_*2*_ central venous oxygen saturation, *SOFA* Sequential Organ Failure Assessment

### Admission measurements

Lactate(adm) was missing in 132 of 821 (16 %) patients, MAP(adm) was missing in 40 of 821 (4.9 %) patients, delta-T(adm) was missing in 284 of 821 (34.6 %) patients and ScvO_2_ was missing in 789 of 821 (96 %) patients.

The number of patients that had a first measured lactate above 2 mmol/l was 378 patients (46 %). A first MAP below 65 mmHg was present in 251 patients (31 %). In these patients, MAP stayed significantly lower over 24 hours compared to patients with a first MAP above 65 mmHg (*p* < 0.001).

Table 2 shows the univariate analysis on both ICU and hospital mortality. Lactate(adm), MAP(adm) and ScvO_2_(adm) were associated with mortality but delta-T(adm) was not. Because of the limited number of ScvO_2_ measurement on admission, ScvO_2_ was not included in the multiple regression analysis using admission data. Table 3 shows that, in the multiple regression analysis, MAP(adm) and lactate(adm) are independently associated with ICU and hospital mortality. Lactate was the strongest predictor as shown by the highest Wald score and highest coefficient. The model fits reasonably well for ICU mortality with a Hosmer-Lemeshow goodness of fit (Chi-square, 13; *p* = 0.10) and ROC area under the curve (AUC) of 0.60. For the hospital mortality, the Hosmer-Lemeshow goodness of fit showed a Chi-square of 23 (*p* = 0.003) and ROC-AUC of 0.64.

**Table 2:** Univariate analysis of circulatory variables on admission and 24-hour means

	ICU mortality	Hospital mortality
Lactate(adm)	1.2 (1.17–1.23)	1.2 (1.17–1.22)
Lactate(24 h)	1.45 (1.39–1.50)	1.45 (1.4–1.50)
MAP(adm)	0.98 (0.98–0.99)	0.986 (0.986–0.991)
MAP(24 h)	0.92 (0.91–93)	0.94 (0.93–0.95)
ScvO_2_(adm)	1.04 (1.0–1.07)	1.0 (0.98–1.04)
ScvO_2_(24 h)	1.03 (1.01–1.05)	1.0 (0.99–1.01)
Delta-T(adm)	0.99 (0.98–1.02)	1.06 (1.04–1.08)
Delta-T(24 h)	1.21 (1.17–1.25)	1.17 (1.13–1.20)
Fluid balance(24 h)	1.00 (1.00–1.00)	1.00 (1.00–1.00)
Dopamine dose(24 h)	1.002 (1.001–1.002)	1.001 (1.001–1.002)
Noradrenaline dose(24 h)	1.03 (1.02–1.04)	1.03 (1.02–1.04)

Values are shown as odds ratios (95 % confidence interval)

*(24 h)* arithmetic mean of all validated measurements during the first 24 hours after admission, *(adm)* at admission, *delta-T* difference between central and forefoot temperature, *ICU* intensive care unit, *MAP* mean arterial pressure, *ScvO*_*2*_ central venous oxygen saturation

**Table 3:** Logistic regression analysis for admission values of MAP, lactate and delta-T

	Coefficient	Wald	OR (95 % CI)	*p* value
ICU mortality				
MAP(adm)	−0.012	54	0.988 (0.985–0.991)	0.001
Lactate(adm)	0.162	173	1.17 (1.15–1.21)	0.001
Delta-T(adm)	−0.07	0.57	0.993 (0.976–1.01)	0.45
Constant	−1.15	72	0.317	0.001
Hospital mortality				
MAP(adm)	−0.008	32	0.992 (0.989–0.995)	0.001
Lactate(adm)	0.159	177	1.17 (1.15–1.20)	0.001
Delta-T(adm)	−0.002	0.083	0.998 (0.983–1.01)	0.77
Constant	−0.894	54	0.409	0.001

*(adm)* at admission, *CI* confidence interval, *delta-T* difference between central and forefoot temperature, *ICU* intensive care unit, *OR* odds ratio, *MAP* mean arterial pressure

### Twenty-four-hour mean values

The univariate analysis (Table 2) showed that all variables were associated with ICU and hospital mortality. Because of the large number of missing values, ScvO_2_ was not included in the consecutive multivariate logistic regression analysis. Twenty-four-hour mean MAP, mean lactate and mean delta-T were entered as independent variables. We show that all three variables were independently related to ICU mortality (Hosmer-Lemeshow goodness of fit Chi-square 98 (*p* = 0.001) and ROC-AUC 0.71) and with hospital mortality (Hosmer-Lemeshow goodness of fit Chi-square 48 (*p* = 0.001) and ROC-AUC 0.68; Table 4). Lactate appeared to be the strongest predictor with the highest Wald score and highest coefficient (Table 4). A repeated analysis including the ScvO_2_ data (*N* = 193) showed that lactate(24 h) was the only independent variable for hospital mortality. APACHE IV significantly predicted hospital mortality (*p* = 0.001). Therefore, we added APACHE IV as a marker for severity of disease in the logistic model but this did not change the relation between the independent variables and hospital mortality (data not shown). Fluid balance, noradrenaline dose and dopamine dose were significantly related to ICU and hospital mortality in a univariate analysis, but in the logistic regression model they were not and were therefore excluded in the backward regression for the final model. We also analyzed the influence of admission chronology by entering the consecutive ICU numbers of the patients, but could not find any relation with outcome.

**Table 4:** Multivariate logistic regression analysis for mean 24-hour measurements

	Coefficient	Wald	OR (95 % CI)	*p* value
ICU mortality				
MAP(24 h)	−0.056	121	0.95 (0.94–0.96)	0.001
Lactate(24 h)	0.28	227	1.32 (1.28–1.37)	0.001
Delta-T(24 h)	0.125	54	1.13 (1.10–1.17)	0.001
Constant	1.06	7.8	2.89	0.005
Hospital mortality				
MAP(24 h)	−0.04	98	0.96 (0.95–0.97)	0.001
Lactate(24 h)	0.30	245	1.34 (1.30–1.40)	0.001
Delta-T(24 h)	0.085	29.9	1.09 (1.06–1.12)	0.001
Constant	0.685	4.8	1.98	0.03

*(24 h)* arithmetic mean of all validated measurements during the first 24 hours after admission, *CI* confidence interval, *delta-T* difference between central and forefoot temperature, *ICU* intensive care unit, *OR* odds ratio, *MAP* mean arterial pressure

**Fig. 1:**
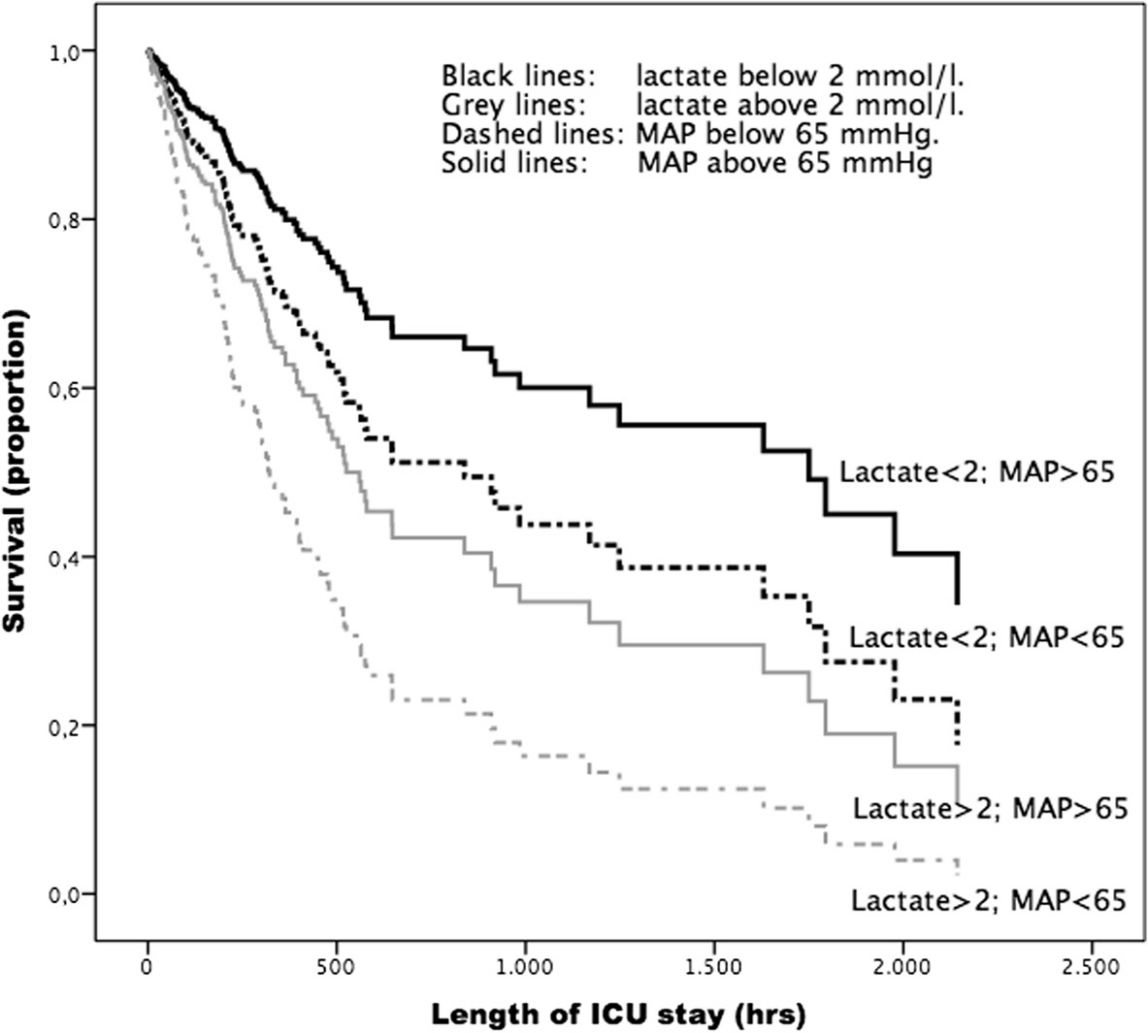
Cox regression model for ICU survival based on the four categories for mean 24-hour values of lactate and MAP. –2Log-likelihood = 1524; Chi-sqare 32, *p* = 0.001 between groups. *ICU* intensive care unit, *MAP* mean arterial pressure

Further analysis of the relative importance of MAP and lactate by a Cox regression prediction model (Fig. 1) shows that MAP(24 h) above 65 mmHg and lactate(24 h) below 2 mmol/l is associated with the lowest mortality and a MAP(24 h) below 65 mmHg and a lactate(24 h) above 2 mmol/l is associated with the highest mortality. The two groups with mean 24-hour lactate levels lower than 2 mmol/l had the best survival during ICU stay.

## Discussion

In this retrospective cohort study including 821 patients with severe sepsis and septic shock we show that both lactate and MAP are, in a multivariate analysis, independently related to ICU and hospital mortality in critically ill patients admitted with severe sepsis or septic shock. Lactate appeared to be the strongest predictor based on the regression coefficients and Wald score. In addition, the Cox regression model shows that the two groups with a low 24-hour mean lactate have the best survival over time. These results suggest that the clinical relevance of lactate, as a resuscitation goal, is of greater importance than MAP. In addition, the present study shows that mean 24-hour delta-T is related to ICU and hospital mortality but that mean ScvO_2_ is not. The number of missing ScvO_2_ measurements was high and may have contributed to this result. These findings are in concordance with Hernandez et al. who showed that only hyperlactatemia was associated with mortality [12, 16], and with the recent analysis of data from the SSC [17]. Our findings add to this knowledge by showing the relatively great importance of lactate in comparison to blood pressure. It was previously shown that patients with a clinically suspected infection and with a lactate value greater than 4 mmol/l in the presence of hypotension had an increased risk of in-hospital mortality [17]. The authors suggest that a trigger for quantitative resuscitation should be at a lactate of 4 mmol/l and maybe even lower at 2 mmol/l. Recent publications on arterial pressure and sepsis advise to maintain a target MAP of at least 65 mmHg (Grade 1C, indicating a strong recommendation with a low level of evidence) [1, 18, 19]. However, the goal for an effective MAP in septic patients is being challenged since studies that strive for a predefined MAP, often by using fluids and vasopressors, did not improve survival [2, 6, 20, 21]. The group of patients with a low lactate and a low MAP needs further study since Hernandez et al. stated that persistent sepsis-induced hypotension without hyperlactatemia is associated with less organ dysfunction and a very low mortality risk [16]. In our study this group has a moderate mortality as shown in the Cox regression model (Fig. 1). The Cox regression shows that both groups with low lactate have the best prognosis in contrast to the two groups with high MAP.

Due to the retrospective study design the results can only be seen as hypothesis generating. In addition, this retrospective cohort analysis does not answer the question whether active treatment of either low blood pressure or elevated lactate will improve survival. Prospective randomized trials will be needed to answer these questions. The present data suggest that future studies concerning resuscitation of patients with severe sepsis and septic shock may focus more on lactate and less on blood pressure. Early lactate clearance is associated with improved outcome [22] and a previous study focusing on lactate normalization indeed showed beneficial effects with decreasing mortality from 43.5 % to 33.9 % [23]. However, which strategies are preferred to reduce lactate levels is still unclear. Skin mottling was independently associated with mortality in a previous study. We confirmed the independent relation of skin temperature with hospital or ICU mortality in our multivariate analysis [24]. However, it is unknown if specific strategies to improve skin perfusion will lead to better outcomes.

Our study has several strengths and limitations. This relatively large cohort had, in the single center design, for all patients a similar basic treatment based on local protocols concerning antibiotics, steroids, mechanical ventilation and feeding. Another strength is the meticulously and prospectively recording of all data as this ICU has used a highly sophisticated and protocolled database for 14 years. There still exists a difference in data collection between MAP, lactate and ScvO_2_, however. MAP is precisely validated at least every hour, but lactate is not routinely measured every hour. As a consequence, when a patient improves, the number of lactate measurements will decrease. By calculating the arithmetic mean of the lactate measurements, we may have overestimated the mean lactate level over the first 24 hours, but this makes our conclusion more likely stronger than weaker. We did not include an analysis of variability of blood pressure or lactate. This was not performed because it would require a more standardized data collection at precise time intervals. A limited number of ScvO_2_ measurements were available (23.5 %) as not all patients had a jugular or subclavian central line. Though this is a clinical reality, it may be true that an analysis with complete hourly ScvO_2_ measurements may have given other results. The U-shaped curve of ScvO_2_ and mortality may play a role as well. We performed an additional analysis with the limited ScvO_2_ data (193 patients). In that analysis lactate was the only independent predictor for hospital mortality (data not shown). It may be possible that ScvO_2_ is of greater importance than is shown in this analysis. Another limitation of the study is that the groups differ not only in blood pressure and lactate level but also in the amount of fluids infused and the use of vasoactive drugs. A large volume of fluids infused is associated with adverse outcome [25]. Sadaka et al. concluded in their resuscitation study that, in patients with more than 6 liters within 24 hours, mortality increased from 42 % to 55–58 %; below 6 liters there are no major differences to be seen [25]. However, in our study it is more logical to suppose that the low blood pressure or high lactate was the reason for extra infusion and vasopressors. We performed an additional regression analysis with fluid balance and dopamine and noradrenaline dose (data not shown). We found that these variables were not independently related to mortality in the multivariate analysis and were excluded in the backward regression. Although, because of its retrospective design, individual resuscitation and circulatory therapy was not standardized in detail, a local protocol for treatment of sepsis and septic shock was available in the complete study. Finally, our study is limited to the first 24 hours after admission to the ICU, but treatment had already started in the emergency room where fluids and antibiotics were initiated.

## Conclusion

We have shown that lactate level on admission and 24-hour mean lactate level, as well as MAP on admission and 24-hour mean MAP are independently related with outcome. In a multivariate regression analysis, 24-hour mean lactate, mean MAP and mean delta-T were independently related to ICU and hospital mortality but lactate was the strongest predictor. The Cox regression model suggests that a 24-hour mean lactate level above 2 mmol/l is more strongly associated with hospital mortality than a mean 24-hour MAP below 65 mmHg.

## Key messages

- Admission MAP and admission lactate are associated with mortality but delta-T is not.
- 24-hour mean lactate, 24-hour mean MAP and 24-hour mean delta-T are associated with ICU and hospital mortality
- 24-hour mean lactate was the strongest predictor for ICU and hospital mortality
- The limited number of ScvO_2_ data hampered final conclusions concerning ScvO_2_
- In a Cox regression, 24-hour mean lactate below 2 mmol/l is more strongly associated with hospital mortality than 24-hour MAP

## Abbreviations

APACHE: Acute Physiology and Chronic Health Evaluation
AUC: Area under the curve
delta-T: Change in temperature between the forefoot and centrally
delta-T(24 h): Mean delta-T over 24 hours
delta-T(adm): Delta-T at admission
ICU: Intensive care unit
Lactate(24 h): Mean lactate over 24 hours
Lactate(adm): Lactate at admission
MAP: Mean arterial pressure
MAP(24 h): Mean arterial pressure over 24 hours
MAP(adm): Mean arterial pressure at admission
OLVG: Onze Lieve Vrouwe Gasthuis
PDMS: Patient Data Management System
ROC: Receiver operator characteristic curve
SSC: Surviving Sepsis Campaign
ScvO_2_: Central venous oxygen saturation
ScvO_2_(24 h): Central venous oxygen saturation over 24 hours
